# Maternal Folic Acid Supplementation, Dietary Folate Intake, and Low Birth Weight: A Birth Cohort Study

**DOI:** 10.3389/fpubh.2022.844150

**Published:** 2022-06-09

**Authors:** Liping Yang, Wenjuan Wang, Baohong Mao, Jie Qiu, Huaqi Guo, Bin Yi, Xiaochun He, Xiaojuan Lin, Ling Lv, Xiaoying Xu, Qing Liu, Yongchun Cao, Yiming Chen

**Affiliations:** ^1^Department of Public Health and Infection Management, Gansu Provincial Hospital of Traditional Chinese Medicine, Lanzhou, China; ^2^Department of Information Administration, Gansu Provincial Maternity and Child Care Hospital, Lanzhou, China; ^3^School of Public Health, Shanghai Jiao Tong University, Shanghai, China; ^4^Department of Operation Management, Gansu Provincial Maternity and Child Care Hospital, Lanzhou, China; ^5^Department of Human Resource, Gansu Provincial Maternity and Child Care Hospital, Lanzhou, China

**Keywords:** folic acid supplementation, dietary folate intake, dietary folate intake, LBW, cohort study

## Abstract

**Objectives:**

To investigate the independent and collective effects of maternal folic acid supplementation or dietary folate intake on the risk of low birth weight (LBW), and to further comprehensively examine the joint associations of folic acid supplementation and dietary folate intake with LBW by various clinical subtypes.

**Design:**

Participants were recruited from Gansu Provincial Maternity and Child Care Hospital. A standardized and structured questionnaire was distributed to collect demographic factors, reproductive and medical history, occupational and residential history, physical activity, and diet. Data on pregnancy-related complications and birth outcomes were extracted from medical records. Unconditional logistic regression models were used to estimate the odds ratio (*OR*) and 95% confidence interval (95% *CI*) for single and joint associations of folic acid supplementation and dietary folate intake with LBW.

**Setting:**

A birth cohort data analysis using the 2010–2012 Gansu Provincial Maternity and Child Care Hospital in Lanzhou, China.

**Participants:**

In total, 9,231 pregnant women and their children were enrolled in the study.

**Results:**

Compared with non-users, folic acid supplementation was associated with a reduced risk of LBW (*OR*: 0.80, 95% *CI*: 0.66–0.97), and the reduced risk was mainly seen for term-LBW (*OR*: 0.59, 95% *CI*: 0.41–0.85), and multiparous-LBW (*OR*: 0.72, 95% *CI*: 0.54–0.94). There were no significant associations between dietary folate intake and LBW, and there was no interaction between folic acid supplement and dietary folate intake on LBW.

**Conclusions:**

Our study results indicated that folic acid supplementation was associated with a reduced risk of LBW, and there was no interaction between folic acid supplements and dietary folate intake on LBW.

## Introduction

Low birth weight (LBW) can increase neonatal mortality and is associated with various infant morbidities (1, 2). Furthermore, it can lead to chronic diseases in later life (3, 4), such as metabolic syndrome, diabetes mellitus type 2, cardiovascular diseases, hypertension, or cancer (5–7), which result in large economic costs in terms of immediate neonatal intensive care, ongoing long-term complex health needs, as well as lost economic productivity. Unfortunately, approximately 16% of infants are born weighing <2,500 g worldwide, which represents more than 22 million LBW babies per year (8). Thus, it is an important public health problem that needs to be solved urgently.

Folate is the generic term for compounds that have vitamin activity similar to that of pteroylglutamic acid and is an anti-anemic and growth factor. Folate functions as a co-enzyme in several single carbon transfers leading to the biosynthesis of purine nucleotides and deoxythymidylic acid essential for DNA and RNA synthesis (9).

Conflicting results have been obtained from epidemiological studies that investigated the association between folic acid intake and the risk of LBW. Eight studies found that folic acid supplementation before and/or during pregnancy reduced the risk of LBW (10–17), but three studies reported no association between folic acid supplementation and LBW (18–20). Only two studies were about the relationship between dietary folate intake and LBW (15, 21). Uno *et al*. found that folate deficiencies are known risk factors for LBW (21), and Rolschau *et al*. found that the effects of supplementing the diet with folic acid given preconceptionally or in the first half of pregnancy in an affluent northern country were a slight increase of birth weight (15). Scholl *et al*. investigated that lower concentrations of serum folate at week 28 were also associated with a greater risk of LBW (22), and Bergen *et al*. found that low folate concentrations (lowest quintile) were associated with birth weight (23). To further comprehensively examine the single and joint associations of folic acid supplementation and dietary folate intake with LBW by various clinical subtypes, we analyzed data from a birth cohort study conducted in Lanzhou, China.

## Materials and Methods

### Study Population

A birth cohort was conducted in 2010–2012 at the Gansu Provincial Maternity and Child Care Hospital, the largest maternity and child care hospital in Lanzhou, China (24). The study population was described previously (24–28), and 10,542 participants completed in-person interviews. Of those, 323 participants were multiple births, 40 were stillbirths, 253 had birth defects, 30 were data missing on birth weight, 1 was <22 weeks in gestational age, and 664 were more than 4,000 g on birth weight, which yielded 9,231 participants who were included in the current analysis. The in-person interview was conducted by trained study interviewers at the hospital. The questionnaire, which was standardized and structured, collected information on demographic factors, reproductive and medical history, environmental factors, and lifestyle factors. Information on birth outcomes and maternal complications was abstracted from the medical records. This study was approved by the Institutional Review Boards of the Gansu Provincial Maternity and Child Care Hospital. All of the women who took the pills provided oral informed consent.

### Low Birth Weight

The gestational age at delivery was calculated in completed weeks from the first day of the last menstrual period. Information on the last menstrual period was extracted from medical records. All self-reported last menstrual period dates were further verified by ultrasound examinations during antenatal care in the hospital. LBW was defined as a birth weight <2,500 g (29), and normal birth weight (NBW) was defined as a birth weight ≥2,500 and ≥4,000 g.

Preterm-LBW infants in our study were defined as infants born LBW between 22 weeks + 0 days and 36 weeks + 6 days of gestation. And the term-LBW infants in our study was defined as infants born LBW between 37 weeks + 0 days and 41 weeks + 6 days of gestation.

Multiparous-LBW infants in our study were defined as infants born LBW whose mothers' parity was more than 1. And nulliparous-LBW infants in our study were defined as infants born LBW whose mothers' parity was 0.

### Folic Acid Supplementation and Dietary Folate Intake

Information on folic acid supplementation was collected for the following two time periods: before conception and during pregnancy. For each time period, the duration and frequency of folic acid supplementation alone and folic acid-containing multivitamins were ascertained. Folic acid supplementation users were defined as those who took folic acid supplementation alone or folic acid-containing multivitamins before conception or during pregnancy. Preconception and pregnancy users were defined as those who took folic acid supplementation alone or folic acid-containing multivitamins before conception and during pregnancy. Only preconception users were defined as those who took folic acid supplementation alone or folic acid-containing multivitamins before conception. Only pregnancy users were defined as those who took folic acid supplementation alone or folic acid-containing multivitamins during pregnancy. Non-users were defined as those who never took folic acid supplementation alone or folic acid-containing multivitamins before conception and/or during pregnancy.

Dietary information was collected *via* a semiquantitative food frequency questionnaire. The daily dietary folate intake was estimated from the frequency of consumption and portion size of food items using the Chinese Standard Tables of Food Consumption (30).

### Statistical Analysis

Depending on intake levels among the total study population, we determined the quartile, denoted as Q1 (<143.24), Q2 (143.24–188.55), Q3 (188.55–254.37), Q4 (≥254.37), and folic acid supplementation was classified into no more than 12 weeks and more than 12 weeks.

Pearson's chi-square tests were used to compare selected characteristics between NBW and LBW. Unconditional logistic regression models were used to estimate the odds ratio (*OR*) and 95% confidence interval (95% *CI*) for single and joint associations of folic acid supplementation and dietary folate intake with LBW and various clinical subtypes. Dose-response relationships (*p* for trend) were calculated based on those categorical levels. In **Tables 2, 3**, we adjusted for maternal age, monthly income per capita, maternal education level, smoking, maternal employment, weight gain during pregnancy, preeclampsia, cesarean section, parity, total energy intake, dietary folate intake, or folic acid supplement. In **Table 4**, we adjusted for maternal age, monthly income per capita, maternal education level, smoking, maternal employ, weight gain during pregnancy, preeclampsia, cesarean section, total energy intake, dietary folate intake, or folic acid supplement. In **Table 5**, we adjusted for maternal age, monthly income per capita, maternal education level, smoking, maternal employ, weight gain during pregnancy, preeclampsia, cesarean section, parity, and total energy intake. All analyses were performed using SAS software, version 9.4 (SAS Institute, Inc., Cary, North Carolina).

## Results

Of the 9,231 singleton live births, 650 were diagnosed with LBW, and 8,581 were NBW. Table 1 shows the distributions of selected characteristics in participants with LBW and NBW births. Women who had LBW births were more likely to be either younger than 25 years old or older than 30 years old, gain <3,000 monthly income per capita (RMB), have less than a college education, smoke, be unemployed during pregnancy, gain less weight during pregnancy, be diagnosed with preeclampsia, be multipara, and adopt cesarean delivery. Distributions of drinking during pregnancy, prepregnancy BMI, and gender of live birth were similar for LBW and NBW births.

**Table 1:** Distributions of selected characteristics in participants with normal birth weight (NBW) and low birth weight (LBW).

**Characteristics**	**NBW (*n* = 8581)**		**LBW (*n* = 650)**		***P*** **-value**
	***n***	**%**	***n***	**%**	
Maternal age					
<25	1919	22.36	195	30.00	<0.001
25 29	3630	42.31	196	30.15	
≥30	3032	35.33	259	39.85	
Monthly income per capita(RMB)					
<3000	4233	54.56	436	73.90	<0.001
≥ 3000	3525	45.44	154	26.10	
Missing	823		60		
Maternal education level					
< College	5131	60.89	516	81.52	<0.001
≥ College	3295	39.11	117	18.48	
Missing	155		17		
Smoking (passive and active)					
No	6923	80.68	489	75.23	<0.001
Yes	1658	19.32	161	24.77	
Drink during pregnancy					
No	8564	99.80	648	99.69	0.391^*^
Yes	17	0.20	2	0.31	
Maternal employ					
No	4050	47.20	409	62.92	<0.001
Yes	4531	52.80	241	37.08	
Pre-pregnancy BMI (kg/m^2^)					
<18.5	1821	21.92	135	22.13	0.078
18.5 23.9	5644	67.93	396	64.92	
≥24.0	843	10.15	79	12.95	
Missing	273		40		
Weight gain during pregnancy (kg)					
<15	2542	30.75	346	59.04	<0.001
15 18.5	2753	33.30	132	22.53	
> 18.5	2972	35.95	108	18.43	
Missing	314		64		
Preeclampsia					
No	8382	97.68	535	82.31	<0.001
Yes	199	2.32	115	17.69	
Parity					
Primipara	6310	73.53	374	57.54	<0.001
Multipara	2271	26.47	276	42.46	
Cesarean section					
No	5428	63.98	290	47.08	<0.001
Yes	3056	36.02	326	52.92	
Missing	97		34		
Gender of live birth					
Male	4427	51.69	326	50.31	0.498
Female	4138	48.31	322	49.69	
Missing	16		2		
Preterm					
No	8177	95.29	153	23.54	<0.001
Yes	404	4.71	497	76.46	

*Calculated by χ^2^ analysis without accounting for missing data*.

* *Fisher's exact test*.

As shown in Table 2, folic acid supplementation was associated with a reduced risk of LBW overall (*OR*: 0.80, 95% *CI*: 0.66–0.97), and the risk of LBW decreased with the increasing duration of folic acid supplementation (*p* for trend <0.001). After stratifying by periods of folic acid supplementation, slightly significant associations were observed for those who took supplements before conception and during pregnancy (*OR*: 0.78, 95% *CI*: 0.59–1.03) or during pregnancy only (*OR*: 0.80, 95% *CI*: 0.65–0.99), and significant duration of dose-responses for folic acid supplements were observed for both (*p* for trend <0.001 and <0.001). However, this pattern is not similar to women who took supplements before conception only at all. Dietary folate intake is not associated with LBW. In addition, there was no interaction between folic acid supplements and dietary folate intake on LBW (Table 3, *p* = 0.223).

**Table 2:** Associations of folate acid supplementation and dietary folate intake with the risk of LBW.

**Folic acid/folate intake duration**	**NBW** **(*n* = 8581)**	**LBW (*n* = 650)**		
		**Cases**	**OR^a^ (*95% CI*)**	**OR^a^(*95% CI*)**
Folic acid supplement				
Non-users	1859	239	1.00	1.00
Users	6722	411	**0.48 (0.40~0.56)**	**0.80 (0.66~0.97)**
≤ 12 weeks	3500	262	**0.58 (0.48~0.70)**	0.84 (0.68~1.03)
>12 weeks	3222	149	**0.60 (0.54~0.67)**	**0.71 (0.55~0.92)**
*P* for trend			<0.001	<0.001
Before conception and during pregnancy	2542	129	**0.40 (0.32~0.49)**	0.78 (0.59~1.03)
<24 weeks	1179	75	**0.50 (0.38~0.64)**	0.85 (0.62~1.17)
≥24 weeks	1363	54	**0.56 (0.48~0.65)**	**0.64 (0.44~0.93)**
*P* for trend			<0.001	<0.001
Before conception only	326	19	**0.45 (0.28~0.73)**	0.71 (0.43~1.18)
≤ 8 weeks	138	10	0.56 (0.29~1.09)	0.73 (0.37~1.45)
>8 weeks	188	9	**0.61 (0.43~0.86)**	0.69 (0.34~1.41)
*P* for trend			0.002	0.031
During pregnancy only	3854	263	**0.53 (0.44~0.64)**	**0.80 (0.65~0.99)**
<12 weeks	1656	138	**0.65 (0.52~0.81)**	0.84 (0.66~1.07)
≥12 weeks	2198	125	**0.67 (0.59~0.74)**	**0.72 (0.56~0.93)**
*P* for trend			<0.001	<0.001
Dietary folate intake (μg/day)				
Before pregnancy				
Q1 <116.01	2090	218	1.00	1.00
Q2 116.01–158.54	2186	126	**0.55 (0.44~0.69)**	0.70 (0.54~1.00)
Q3 158.54–220.88	2134	168	**0.87 (0.78~0.97)**	1.02 (0.91~1.16)
Q4 ≥220.88	2171	138	**0.85 (0.79~0.91)**	0.96 (0.86~1.06)
*P* for trend			<0.001	0.255
During pregnancy				
Q1 <149.81	2086	224	1.00	1.00
Q2 149.81–197.59	2125	151	**0.66 (0.53~0.82)**	1.01 (0.79~1.29)
Q3 197.59–263.67	2144	138	**0.77 (0.69~0.86)**	0.96 (0.84~1.10)
Q4 ≥263.67	2226	137	**0.83 (0.77~0.90)**	1.02 (0.91~1.15)
*P* for trend			<0.001	0.133

*Odds ration (OR)^a^ univariate analyses*.

OR^b^ *adjusted for maternal age, monthly income per capita, maternal education level, smoking, maternal employ, weight gain during pregnancy, preeclampsia, cesarean section, parity, total energy intake, dietary folate intake, or folic acid supplement*.

**Table 3:** Odds ration (95% *CI*) of LBW by joint effects of folic acid supplement and dietary folate intake.

**Dietary folate intake levels (μg/day)**	**Folic acid supplementation non-users**		**Folic acid supplementation users**		***P*** **for interaction**
	**case/controls**	***OR^d^*** **(*95% CI*)**	**case/controls**	***OR^d^*** **(*95% CI*)**	
Q1 <143.24	98/585	1.00	124/1480	0.82 (0.59~1.15)	0.223
Q2 143.24–188.55	42/347	0.95 (0.76~1.18)	103/1822	0.91 (0.80~1.03)	
Q3 188.55–254.37	48/386	0.94 (0.83~1.06)	100/1779	0.91 (0.84~0.98)	
Q4 ≥254.37	51/541	0.97 (0.87~1.09)	84/1641	0.96 (0.88~1.04)	

OR^d^ *Adjusted for maternal age, education level, monthly income per capita, smoking, employment, weight gain during pregnancy, preeclampsia, parity, cesarean section, and total energy intake*.

We then analyzed the data separately for term-LBW and preterm-LBW (Table 4). The protective effect of folic acid supplementation was increased on term-LBW (*OR*: 0.59, 95% *CI*: 0.41–0.85), but it was not presented at preterm-LBW (*OR*: 0.90, 95% *CI*: 0.72–1.12), and the similar associations were observed for women who took supplements before conception and during pregnancy (*OR*: 0.40, 95% *CI*: 0.23–0.70; *p* for trend <0.001) or during pregnancy only (*OR*: 0.67, 95% *CI*: 0.45–0.98; *p* for trend <0.013). For dietary folate intake, there were no significant associations between term-LBW and preterm-LBW.

**Table 4:** Associations of folate acid supplementation and dietary folate intake with the risk of term-LBW and preterm-LBW.

**Folic acid/folate intake duration**	**NBW** **(*n*= 8581)**	**Term-LBW (37≥weeks)**		**Preterm-LBW (<37 weeks)**	
		**Cases (*n*= 153)**	***OR^b^*** **(*95% CI*)**	**Cases (*n*= 497)**	***OR^b^*** **(*95% CI*)**
Folic acid supplement					
Nonusers	1859	60	1.00	179	1.00
Users	6722	93	**0.59 (0.41~0.85)**	318	0.90 (0.72~1.12)
≤ 12 weeks	3500	60	**0.65 (0.44~0.96)**	202	0.92 (0.73~1.16)
>12 weeks	3222	33	**0.71 (0.55~0.90)**	116	0.90 (0.78~1.04)
*P* for trend			<0.001		<0.001
Before conception and during pregnancy	2542	23	**0.40 (0.23~0.70)**	106	0.96 (0.71~1.30)
<24 weeks	1179	15	0.54 (0.29~1.01)	60	0.99 (0.69~1.41)
≥24 weeks	1363	8	**0.26 (0.11~0.59)**	46	0.92 (0.75~1.13)
*P* for trend			<0.001		0.002
Before conception only	326	6	0.71 (0.29~1.70)	13	0.70 (0.38~1.30)
During pregnancy only	3854	64	**0.67 (0.45~0.98)**	199	0.87 (0.69~1.11)
<12 weeks	1656	29	0.65 (0.41~1.03)	109	0.91 (0.69~1.20)
≥12 weeks	2198	35	0.81 (0.64~1.02)	90	0.88 (0.76~1.02)
*P* for trend			0.013		0.001
Dietary folate intake (μg/day)					
Before pregnancy					
Q1 <116.01	2090	48	1.00	170	1.00
Q2 116.01–158.54	2186	23	0.61 (0.36~1.03)	103	0.72 (0.54~0.99)
Q3 158.54–220.88	2134	41	1.08 (0.86~1.36)	127	1.00 (0.87~1.15)
Q4 ≥220.88	2171	41	1.07 (0.89~1.29)	97	0.91 (0.81~1.03)
*P* for trend			0.352		0.049
During pregnancy					
Q1 <149.81	2086	46	1.00	178	1.00
Q2 149.81–197.59	2125	29	0.88 (0.53~1.47)	122	1.03 (0.78~1.36)
Q3 197.59–263.67	2144	37	1.18 (0.92~1.51)	101	0.88 (0.75~1.04)
Q4 ≥263.67	2226	41	1.14 (0.92~1.42)	96	0.97 (0.84~1.12)
*P* for trend			0.170		0.009

OR^b^ *adjusted for maternal age, monthly income per capita, maternal education level, smoking, maternal employ, weight gain during pregnancy, preeclampsia, cesarean section, total energy intake, dietary folate intake, or folic acid supplement*.

Depending on parity, we divided LBW into nulliparous-LBW and multiparous-LBW (Table 5). A significantly protective effect on multiparous-LBW was observed among folic acid supplementation users (*OR*: 0.72, 95% *CI*: 0.54–0.94), but folic acid supplementation was not related to nulliparous-LBW (*OR*: 0.88, 95% *CI*: 0.68–1.13). We further analyzed multiparous-LBW by periods of folic acid supplementation, the protective effect was significantly increased before conception and during pregnancy (*OR*: 0.55, 95% *CI*: 0.36–0.83), but not during pregnancy only (*OR*: 0.76, 95% *CI*: 0.57–1.02) or before conception only (*OR*: 0.57, 95% *CI*: 0.26–1.27), and a significant duration of dose-responses for folic acid supplementation was observed before conception and during pregnancy (*p* for trend < 0.001), and during pregnancy only (*p* for trend <0.001). For dietary folate intake, there were no significant associations with nulliparous-LBW and multiparous-LBW.

**Table 5:** Associations of folate acid supplementation and dietary folate intake with the risk of nulliparous-LBW and multiparous-LBW.

**Folic acid/folate intake duration**	**NBW** **(*n*= 8581)**	**Nulliparous –LBW**		**Multiparous -LBW**	
		**Cases(*n*= 374)**	***OR^c^*** **(*95% CI*)**	**Cases(*n*= 276)**	***OR^c^*** **(*95% CI*)**
Folic acid supplement					
Nonusers	1859	122	1.00	117	1.00
Users	6722	252	0.88 (0.68~1.13)	159	**0.72 (0.54~0.94)**
≤ 12 weeks	3500	154	0.90 (0.69~1.18)	108	0.77 (0.58~1.04)
>12 weeks	3222	98	0.94 (0.80~1.10)	51	**0.74 (0.61~0.89)**
*P* for trend			<0.001		<0.001
Before conception and during pregnancy	2542	88	0.98 (0.70~1.38)	41	**0.55 (0.36~0.83)**
<24 weeks	1179	49	1.01 (0.68~1.50)	26	0.67 (0.42~1.07)
≥24 weeks	1363	39	0.98 (0.78~1.22)	15	**0.61 (0.45~0.83)**
*P* for trend			0.020		<0.001
Before conception only	326	12	0.85 (0.45~1.62)	7	0.57 (0.26~1.27)
During pregnancy only	3854	152	0.88 (0.67~1.15)	111	0.76 (0.57~1.02)
<12 weeks	1656	79	0.90 (0.66~1.24)	59	0.79 (0.56~1.12)
≥12 weeks	2198	73	0.90 (0.76~1.06)	52	**0.82 (0.68~0.98)**
*P* for trend			0.008		<0.001
Dietary folate intake (μg/day)					
Before pregnancy					
Q1 <116.01	2090	123	1.00	95	1.00
Q2 116.01–158.54	2186	75	0.69 (0.50~0.95)	51	0.74 (0.50~1.09)
Q3 158.54–220.88	2134	98	1.01 (0.87~1.18)	70	1.04 (0.87~1.24)
Q4 ≥220.88	2171	78	0.90 (0.79~1.03)	60	1.05 (0.90~1.22)
*P* for trend			0.171		0.894
During pregnancy					
Q1 <149.81	2086	117	1.00	107	1.00
Q2 149.81–197.59	2125	87	1.00 (0.73~1.39)	64	0.96 (0.67~1.38)
Q3 197.59–263.67	2144	91	1.02 (0.86~1.22)	47	0.86 (0.70~1.06)
Q4 ≥263.67	2226	79	1.01 (0.87~1.18)	58	1.03 (0.87~1.23)
*P* for trend			0.308		0.135

OR^c^ *adjusted for maternal age, education level, monthly income per capita, smoking, employment, weight gain during pregnancy, preeclampsia, cesarean section, total energy intake, dietary folate intake, or folic acid supplement*.

## Discussion

Our study results indicated that folic acid supplementation was associated with a reduced risk of LBW, term-LBW, and multiparous-LBW, with those risks decreasing with increasing duration of folic acid supplementation. After stratifying by periods of folic acid supplementation, similar patterns were observed for those who took supplements before conception and during pregnancy or pregnancy only. There was no interaction between folic acid supplement and dietary folate intake on LBW.

The occurrence of LBW is a highly complex biologic process, and the precise protective mechanism of folic acid is still unknown. The epigenome is particularly susceptible during the early stages of embryogenesis (31), folate may cause epigenetic modifications resulting in increased placental and fetal growth patterns (32, 33). In addition, folate may indirectly influence fetal growth by modulating placental growth and development (34, 35), and folate plays a critical role in protein and DNA synthesis (36, 37).

Earlier epidemiological research investigating the associations between folic acid supplements and the risk of LBW has provided ambiguous results. In Europe, five studies (10–14) based on cohort studies indicated that folic acid supplementation was associated with birth weight and one cohort study (15) indicated that the effects of supplementing the diet with folic acid given preconceptionally or in the first half of pregnancy were a slight increase of birth weight. Timmermans *et al*. found that preconception start of folic acid was associated with a decreased risk of LBW, and the start of folic acid supplementation after pregnancy recognition was also associated with a decrease of having a child with LBW (10). Pastor-Valero *et al*. thought periconceptional use of folic acid supplements > 1 mg/day may entail a risk of decreased birth weight (13), and Papadopoulou *et al*. indicated that high daily doses of supplementary folic acid in early-to-mid pregnancy may be protective for LBW (12). In addition, Bergen *et al*. found that low folate concentrations and erythrocyte folic acid were associated with birth weight (23). However, a case control study indicated that there was no significant reduction in the rate of LBW in pregnant women with early or late onset pre-eclampsia after folic acid supplementation (20). In Japan, only one study indicated that lower dietary intake of protein, iron, and folic acid are known risk factors for LBW (21). In the United States, Martinussen *et al*. found that there were no significant associations between folic acid supplementation and LBW (19), but Scholl *et al*. indicated that lower concentrations of serum folate at week 28 were also associated with a greater risk of preterm delivery and LBW (22). In China, Li *et al*. indicated that statistically significant reductions in the risk were evident in women who used folic acid peri- or postconception, but not in those who took folic acid preconception (16). Liu *et al*. found that the risk of LBW among pregnant women who did not take folic acid during periconception was 1.30 times higher than those who took folic acid (17), but Yang *et al*. found that folic acid supplementation was not associated with birth weight (18).

The different recommendations about folic acid supplements and dietary pattern among international entities may contribute to the conflicting results. To prevent neural tube defects and other congenital anomalies, more than forty seven countries have recommended taking folic acid supplements in the periconceptional period (38) based on two randomized trials by the British Medical Research Council in 1991 and Hungarian National Institute of Hygiene in 1992 (39, 40). In Europe, no folic acid fortification is required and only voluntary fortification is permitted (41). In North America, folic acid fortification is mandatory in grain products. In China, women were advised to supplement folic acid at least 4 weeks before conception and throughout the pregnancy. Other countries/regions, such as Singapore and Taiwan, emphasize the importance of a healthy diet without the need for supplementation, while Slovenia, Sweden, and Hong Kong published e-leaflets for the general public with detailed information about folate healthy diet during pregnancy (38). In addition to folate, variations in study populations, the time for initiating supplementation of folic acid, and the dosing of use of folic acid may also contribute to the conflicting results.

Our study discovered significant dose-response for supplement duration for those who took supplements before conception and during pregnancy or during pregnancy only, indicating that the risk of LBW, term-LBW, and multiparous-LBW decreased with increasing duration of folic acid supplementation. In China, Li *et al*. (16) found that the trend relative risks significantly decreased as compliance with folic acid use increased. However, the significant dose-response for the duration of supplementation was not shown, and other previous studies also did not explore this association. This result was important for preventing LBW, and suggested that starting folic acid supplementation should be done earlier in pregnancy and continued for at least 12 weeks.

To our knowledge, this is the first study investigating the associations of term-LBW and preterm-LBW with folic acid supplementation as well as the associations of nulliparous-LBW and multiparous-LBW with folic acid supplementation. Significant associations were observed for term-LBW and multiparous-LBW but not for preterm-LBW or nulliparous-LBW, which indicated that they may have different etiological profiles, and the biologic processes should be further studied.

Actually, there are some limitations to the current study. First, the study participants were predominantly from Lanzhou, so generalizability of our results to other populations with quite different demographic characteristics may not be appropriate. Second, dietary folate in a combination with other micronutrients could potentially confound our results. In models b, c, and d, we have adjusted for total energy, which could control this problem effectively. Third, although we have adjusted for many important confounding factors, we cannot rule out the potential for residual confounding. Since information on folic acid supplementation and dietary folate intake was based on self-reported data, there existed a recall bias. However, during the period of questionnaire design, field investigation, and information input, there were enough professionals who undertook the quality control, ensuring the accuracy of information. In addition, a study has already suggested that there is a strong correlation between self-reported folate intake and serum folate concentrations during pregnancy (22).

## Conclusion

Our study results indicated that folic acid supplementation was associated with a reduced risk of LBW, term-LBW, and multiparous-LBW, with those risks decreasing with increasing duration of folic acid supplementation. After stratifying by time periods of folic acid supplementation, similar patterns were observed for those who took supplements before conception and during pregnancy or during pregnancy only. And there was no interaction of folic acid supplement and dietary folate intake on LBW.

## Data Availability Statement

The original contributions presented in the study are included in the article/supplementary material, further inquiries can be directed to the corresponding authors.

## Ethics Statement

The studies involving human participants were reviewed and approved by Human Investigation Committees at the GPMCCH. The patients/participants provided their written informed consent to participate in this study. Written informed consent was obtained from the individual(s) for the publication of any potentially identifiable images or data included in this article.

## Author Contributions

QL and JQ designed the birth cohort study. LY, WW, and BM analyzed data and wrote the article. LY, WW, BM, BY, XH, XL, LL, XX, YCh, and YCa conducted this research. YCh and YCa had primary responsibility for the final content. All authors read and approved the final manuscript.

## Funding

This study was financially supported by BiosTime Maternal and Child Nutrition Health Research Projects of China CDC Maternal and Child Health Center (No. 2019FYH002), in part supported by Gansu Provincial Science and Technology Department Grant (No. 21JR1RA043), the Health Research Projects (No. GSWSKY-2019-98), and the Key Research and Development Program (No. 20YF8WA095). The funders had no role in the design, analysis, or writing of this article.

## Conflict of Interest

The authors declare that the research was conducted in the absence of any commercial or financial relationships that could be construed as a potential conflict of interest.

## Publisher's Note

All claims expressed in this article are solely those of the authors and do not necessarily represent those of their affiliated organizations, or those of the publisher, the editors and the reviewers. Any product that may be evaluated in this article, or claim that may be made by its manufacturer, is not guaranteed or endorsed by the publisher.

## Abbreviations

LBW: Low birth weight
NBW: Normal birth weight
GPMCCH: Maternity and Child Care Hospital
OR: Odds ratio
CI: Confidence interval.
